# Ethnic and seasonal variations in FGF-23 and markers of chronic kidney disease–mineral and bone disorder

**DOI:** 10.1093/ckj/sfae188

**Published:** 2024-06-20

**Authors:** Hulya Taskapan, Sara Mahdavi, Antonio Bellasi, Salome Martin, Saeeda Kuvadia, Anfal Patel, Berkay Taskapan, Paul Tam, Tabo Sikaneta

**Affiliations:** Research Department, Kidney Life Sciences Institute, Toronto, Canada; Harvard T.H. Chan School of Public Health, Boston, USA; Department of Medicine, University of Canada, Toronto, Canada; Department of Nephrology, The Scarborough Health Network, Toronto, Canada; Department of Nephrology, Ente Ospedaliere Cantonale, Lugano, Switzerland; Department of Nephrology, The Scarborough Health Network, Toronto, Canada; Department of Nephrology, The Scarborough Health Network, Toronto, Canada; Department of Medicine, University of Canada, Toronto, Canada; Department of Nephrology, The Scarborough Health Network, Toronto, Canada; Research Department, Kidney Life Sciences Institute, Toronto, Canada; Research Department, Kidney Life Sciences Institute, Toronto, Canada; Department of Medicine, University of Canada, Toronto, Canada; Research Department, Kidney Life Sciences Institute, Toronto, Canada; Department of Medicine, University of Canada, Toronto, Canada; Department of Nephrology, The Scarborough Health Network, Toronto, Canada

**Keywords:** CKD-MBD, epidemiology, ethnicity, FGF-23, pre-dialysis

## Abstract

**Background:**

Fibroblast growth factor 23 (FGF-23) and other markers of chronic kidney disease–mineral and bone disorder (CKD-MBD) provide valuable insights into disease processes, treatment options and patient prognosis. However, limited research has explored potential associations with ethnicity or season, particularly in multi-ethnic populations residing in high-latitude regions.

**Methods:**

We evaluated CKD-BMD markers in a diverse cohort of CKD patients, who were participants of The CANADIAN AIM to PREVENT (the CAN AIM to PREVENT) study. FGF-23, calcium, phosphate, 25-hydroxyvitamin D (25-OHD) and intact parathyroid hormone (iPTH) in 1234 participants with pre-dialysis CKD (mean estimated glomerular filtration rate: 41.8 ± 14.3 mL/min) were analyzed. Mixed-effects general linear regression models adjusted for demographic and biological factors were used to compare repeated measurements across patient groups categorized by ethnicity (East Asian, White, South Asian, Black, Southeast Asian) and seasons.

**Results:**

Compared with other groups, White participants exhibited 8.0%–18.5% higher FGF-23 levels, Black participants had 0.17–0.32 mg/dL higher calcium levels, White participants had 10.0%–20.1% higher 25-OHD levels, South Asian participants had 7.3%–20.1% lower 25-OHD levels and Black participants had 22.1–73.8% higher iPTH levels, while East Asian participants had 10.7%–73.8% lower iPTH levels. Seasonal variations were also observed. FGF-23 levels were 11.9%–15.5% higher in summer compared with other seasons, while calcium levels were 0.03–0.06 mg/dL lower in summer. 25-OHD levels were 5.6%–10.6% higher in summer and autumn compared with other seasons.

**Conclusions:**

This study shows that FGF-23 and CKD-MBD markers in a Canadian pre-dialysis CKD cohort vary independently by ethnicity and season. Further research is needed to understand the reasons and clinical significance of these findings.

## Graphical Abstract

**Graphical Abstract:**
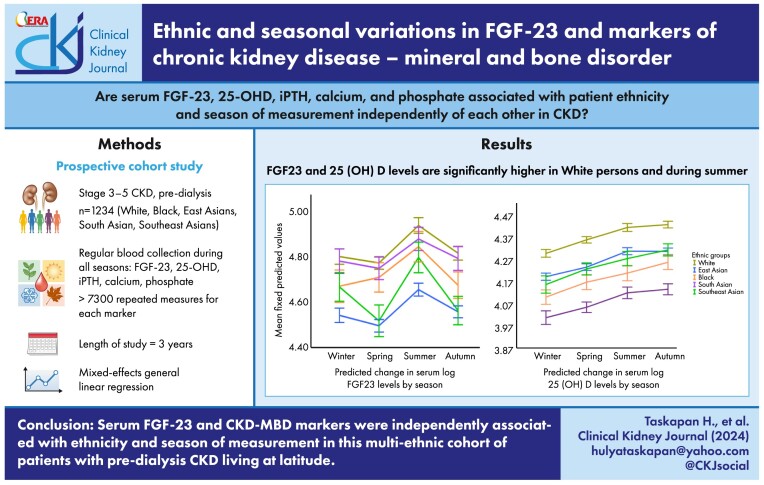

KEY LEARNING POINTS **What was known:**

- Previous studies have hinted at potential variations in fibroblast growth factor 23 (FGF-23) and other chronic kidney disease–mineral and bone disorder (CKD-MBD) markers based on ethnicity and season, but these investigations were limited; they often focused on a small number of markers, included ethnically homogenous populations, or were conducted in lower latitude regions.
- Given the growing multi-ethnic populations in high-latitude countries, and the importance of FGF-23 and CKD-MBD markers in disease understanding and prognosis, further research is warranted.

**This study adds:**

- This study unearthed several key findings regarding FGF-23, CKD-MBD markers, and their relationship to ethnicity and season as follows.
- Seasonal influence on FGF-23: for the first time, this study revealed a significant seasonal variation in FGF-23 levels. Participants had 11.9%–15.5% higher FGF-23 levels in the summer compared with other seasons.
- Ethnic differences in FGF-23: the study confirmed ethnicity-based disparities in FGF-23, with White participants having 8%–18.5% higher levels compared with other ethnicities.
- 25-Hydroxyvitamin D (25-OHD) and ethnicity paradox: interestingly, the study found that despite higher vitamin D supplementation, South Asian participants had significantly lower 25-OHD levels compared with other ethnicities. This highlights possibility of alternative vitamin D metabolism within this ethnic group.

**Potential impact:**

- The study's findings pave the way for future research that could significantly improve our understanding and management of CKD-MBD. The following are two key areas for exploration.
- Unraveling the regulatory mechanisms: future studies can delve into the intricate interplay between seasonal factors like sunlight exposure, temperature and physical activity, alongside ethnicity-specific factors, to understand how they regulate FGF-23 concentrations. This could involve pinpointing the underlying mechanisms by which these factors influence FGF-23 production or clearance within the body.
- Clinical significance of variations: the observed variations in FGF-23 and other CKD-MBD markers warrant further investigation into their clinical impact. Future studies could examine whether these variations, particularly those observed for FGF-23 (11.9%–15.5% difference), translate into a meaningful difference in patient outcomes for pre-dialysis CKD patients. This could involve analyzing if these variations are associated with a higher risk of complications or poorer prognosis. By establishing the clinical significance of these variations, we can potentially refine treatment strategies and improve patient care.

## INTRODUCTION

Chronic kidney disease (CKD) disrupts the intricate interplay between several hormones and minerals that maintain healthy bones and blood chemistry [1–5]. Fibroblast growth factor 23 (FGF-23) emerges as a key player early in CKD, acting to curb rising phosphate levels by increasing its excretion through the kidneys [1–5]. However, this action has consequences. FGF-23 also suppresses the production of 1,25-hydroxyvitamin D [1,25-(OH)_2_D], a hormone crucial for calcium absorption [1–5]. This can lead to subtle decreases in calcium, which in turn stimulates parathyroid hormone (PTH) production [1–5]. As a result, calcium and phosphate levels are often maintained within a normal range in early CKD stages [6–8]. However, this delicate balance can tip over in later stages of CKD. Hyperphosphatemia and subsequently, hypocalcemia become more frequent as the disease progresses [6–8]. These abnormalities, collectively termed CKD–mineral and bone disorder (CKD-MBD), are significant risk factors for complications and death in CKD patients [9–11]. Therefore, diagnosing, evaluating, preventing and treating CKD-MBD is a crucial aspect of managing CKD.

Current guidelines for managing CKD-MBD recommend monitoring calcium, phosphate, 25-hydroxyvitamin D (25-OHD) and PTH levels [12, 13]. Notably, these guidelines do not yet include measuring FGF-23, partly due to the lack of standardized reference ranges for different assays [12, 13]. Additionally, ethnicity and seasonal variations are not considered when interpreting these test results, despite evidence suggesting their influence on some CKD-MBD markers [14–22]. For example, studies have shown lower 25-OHD and FGF-23 levels, and higher PTH levels in Black populations compared with White populations [14–18]. Similarly, higher 25-OHD levels are observed during summer months [19–22]. These prior studies, however, had limitations. They often focused on only a few CKD-MBD markers, included small patient groups, or had methodological drawbacks like retrospective designs or limited geographic locations [14–22]. Additionally, some studies focused solely on patients undergoing dialysis or compared only Black and White (or non-Black) ethnicities [14–22].

The goal of this study was to assess if ethnicity and season is associated with FGF-23 and other CKD-MBD markers, in Toronto (latitude 43.7°), Canada, in a large, multi-ethnic cohort of pre-dialysis CKD patients.

## MATERIALS AND METHODS

The CANADIAN AIM to PREVENT (the CAN AIM to PREVENT) study, described elsewhere [23], was an investigator-initiated prospective open observational cohort study of 2254 patients followed in three predialysis clinics in Toronto from 2010 to 2015. The primary objective was to predict dialysis progression in relation to markers of inflammation. It was registered at http://www.clinicaltrials.gov (#NCT01974713). Patients were invited by their usual nephrologist to participate in this study. After obtaining signed informed consent, demographic information and clinical histories were recorded. Information on medication use, vital signs and laboratory values including serum concentrations of FGF-23, calcium, phosphate, 25-OHD and iPTH were obtained at baseline and every 6 months for up to 3 years. Plasma samples for FGF-23 measurements were batched and sent to a centralized research laboratory in San Clemente, CA, USA every 6 months. An enzyme-linked immunosorbent assay using reagents from Immutopics (San Clemente, CA, USA) targeted against the carboxy-terminus of FGF-23 (thereby measuring both intact and carboxy-terminal fragments) was used to measure FGF-23 levels. Calcium, phosphate, 25-OHD, iPTH and all other routine renal laboratory tests were performed using standard assays in commercial provincial laboratories. Calcium concentrations were corrected for serum albumin levels. Glomerular filtration rates were estimated (eGFR) using the 2009 creatinine-based Chronic Kidney Disease Epidemiology Collaboration equation. 25-OHD was categorized as deficient if <20 ng/mL, insufficient if 20–30 ng/mL and sufficient if ≥30 ng/mL. Patients were censured at initiation of renal replacement or erythropoietin-stimulating therapy, withdrawal of consent, transfer to another institution, loss to follow-up or death [24].

After need for additional written consent was waived by an Ethics Review Board, the subset of all 1852 patients at the largest and most ethnically diverse of the three centres (Scarborough) was further characterized in 2020 by contacting participants (or previously designated contacts if necessary) by telephone to ask their ethnicity. If they could not be reached their clinical records were reviewed for documentation of this information. Ethnicity was categorized as Aboriginal, Black, East Asian, Hispanic, Middle East/Arabic, South Asian, Southeast Asian, White, other/multiracial or unknown/did not answer. We then excluded those ethnic groups with fewer than 20 people and those categorized as other/multiracial as they were considered too small or ambiguous to include in the statistical analyses. Patients without FGF-23 measurements at the first study visit were also excluded as they were considered to have incomplete study data.

### Statement of ethics

Ethics review board approval was granted to conduct and the protocol was registered at http://www.clinicaltrials.gov (#NCT01974713). As a *post hoc* analysis of data acquired in the CAN AIM to PREVENT, no additional ethics review board approval was sought for the current study. Written informed consent was obtained from participants (or their parent/legal guardian/next of kin) to participate in the CAN AIM to PREVENT. Additional written informed consent was not obtained for this *post hoc* analysis. The study was conducted ethically and in accordance with the World Medical Association Declaration of Helsinki.

### Statistical methods

All data were analyzed in Stata (StataCorp. 2023; Stata Statistical Software: Release 17; StataCorp LP, College Station, TX, USA). Descriptive statistics were reported using means, medians and frequencies as appropriate. Categorical variables were compared using chi-square analysis (*P *< .05 considered significant). To achieve symmetric distributions, FGF-23, 25-OHD, iPTH and urine protein/creatinine were naturally log-transformed. Kruskal–Wallis testing was used to compare baseline characteristics and laboratory values across ethnic groups.

Mixed-effects general linear regression was used to predict changes in repeated measures of FGF-23, calcium, phosphate, 25-OHD and iPTH. Subject identification number was the only random effect and an autoregressive structure order one (AR1) was assumed in all models. Each dependent variable was fitted in relation to the fixed effects of age, gender, body surface area, visit number (as a proxy for time), eGFR, history of diabetes mellitus, use of vitamin D supplements, urine protein/creatinine ratio and ethnicity or season of measurement, and serum FGF-23, calcium, phosphate, 25-OHD or iPTH as appropriate. Differences in naturally log-transformed variables between ethnic or season groups (log-level regression) were reported as percentages using the anti-log of model-predicted coefficients, [exp(B) – 1] × 100.

## RESULTS

This study investigated the influence of ethnicity and season on serum concentrations of FGF-23 and other CKD-MBD markers in a multi-ethnic cohort of 1234 participants with pre-dialysis CKD in Toronto, Canada.

**Figure 1:**
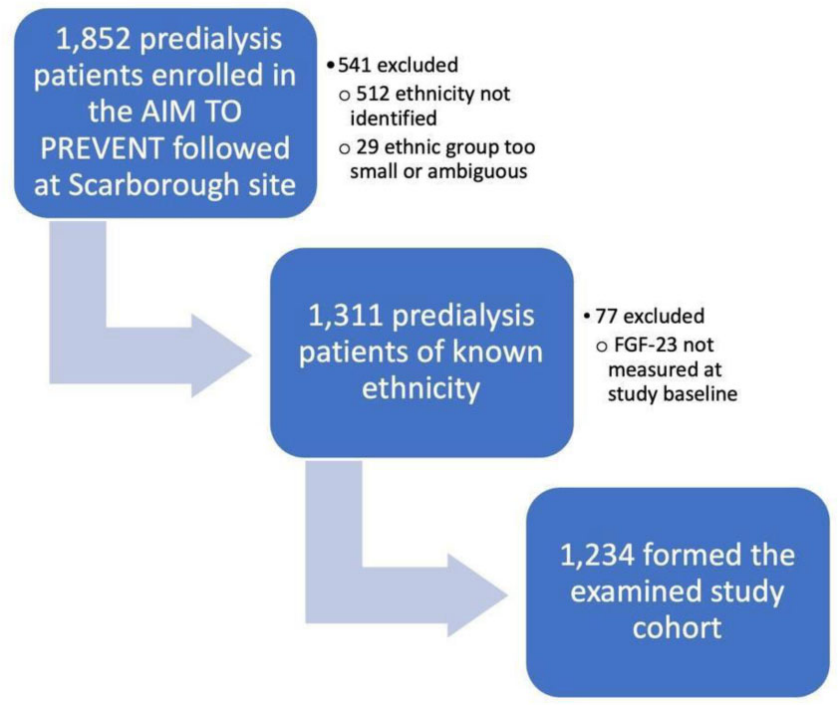
Derivation of the study cohort.

Information about ethnicity was available from 1340 (72.4%) of 1852 Toronto CKD Clinic participants of the CAN AIM to PREVENT (Fig. 1). Inability to reach the participants or contacts by telephone, and lack of documentation in the patient records were the primary reasons for failure to obtain this information. A further 29 patients were excluded as their ethnic group had fewer than 20 persons or were categorized as other/multiracial. Another 77 patients were then excluded as they did not have FGF-23 measurements at their first clinic visit.

The resultant study cohort of 1234 participants attended 7418 study visits where 7398 measurements for FGF-23, and 7329 for calcium, 7393 for phosphate, 7371 for 25-OHD and 7311 for iPTH were conducted. People of East Asian ethnicity formed the largest ethnic group [437 persons (35%)], followed by White [406 (32.9%)], South Asian [169 (13.7%)], Black [128 (10.4%)] and Southeast Asian [94 (7.6%)] persons. Their baseline characteristics are presented in Table 1.

**Table 1:** Baseline characteristics by ethnic groups.

	Total (*n* = 1234)	White (*n* = 406)	East Asian (*n* = 437)	Black (*n* = 128)	South Asian (*n* = 169)	Southeast Asian (*n* = 94)	*P*-value
Age (years)	69.3 ± 12.2	71.3 ± 11.5	71.1 ± 11.8	63.6 ± 12.8	65.6 ± 12.7	66.1 ± 11.1	<.0001^a,b,c,d,e,f^
% Male	66	63	67	59	72	66	.168
% with DM	47	41	44	48	63	62	<.0001
Calcium (mg/dL)	9.2 ± 0.5	9.2 ± 0.4	9.2 ± 0.5	9.3 ± 0.6	9.2 ± 0.5	9.3 ± 0.5	.005^b,e,i^
Phosphate (mg/dL)	3.6 ± 0.6	3.6 ± 0.6	3.6 ± 0.6	3.5 ± 0.6	3.7 ± 0.6	3.6 ± 0.7	.127
25-OHD (ng/mL)	27.1 ± 12.7	30.8 ± 13.8	27.0 ± 11.8	24.0 ± 11.9	21.7 ± 11.2	25.2 ± 9.9	<.0001^a,b,c,d,e,f,j^
Natural log 25-OHD	3.2 ± 0.5	3.3 ± 0.5	3.2 ± 0.5	3.1 ± 0.5	2.9 ± 0.6	3.1 ± 0.5	
PTH (pg/mL)	58 ± 48	59 ± 45	46 ± 30	84 ± 85	69 ± 51	52 ± 35	<.0001^a,b,c,e,f,h,j^
Natural log PTH	3.9 ± 0.6	3.9 ± 0.5	3.7 ± 0.5	4.2 ± 0.6	4.0 ± 0.6	3.8 ± 0.6	
FGF-23 (pmol/L)	149.8 ± 132.7	179.1 ± 180.4	125.7 ± 88.3	155.6 ± 113.0	151.1 ± 112.6	124.5 ± 85.4	<.0001^a,d,e,f,j^
Natural log FGF-23	4.8 ± 0.7	4.9 ± 0.7	4.7 ± 0.6	4.8 ± 0.7	4.8 ± 0.7	4.6 ± 0.6	
BSA (m^2^)	1.8 ± 0.2	1.9 ± 0.2	1.7 ± 0.2	1.9 ± 0.2	1.8 ± 0.2	1.8 ± 0.2	<.0001^a,c,d,e,f,g,h,i^
eGFR (mL/min/1.73 m^2^)	41.8 ± 14.3	38.5 ± 15.3	40.9 ± 15.1	43.0 ± 20.1	38.7 ± 16.5	37.05 ± 15.2	.021^a,b,i^
Urine protein/creatinine (mmol/L)	68.80 ± 129.37	65.53 ± 150.59	80.74 ± 137.48	113.71 ± 219.14	112.42 ± 255.34	121.51 ± 223.74	<.0001^a,c,d,i^
% on vitamin D supplements	23	24	21	20	28	18	.741

^a^White vs East Asian; ^b^White vs Black; ^c^White vs South Asian; ^d^White vs Southeast Asian; ^e^East Asian vs Black; ^f^East Asian vs South Asian; ^g^East Asian vs Southeast Asian; ^h^Black vs Southeast Asian; ^i^South Asian vs Black; ^j^South Asian vs Southeast Asian.

DM, diabetes mellitus; BSA, body surface area.

### Multi-ethnic variations in CKD-MBD markers

**Figure 2:**
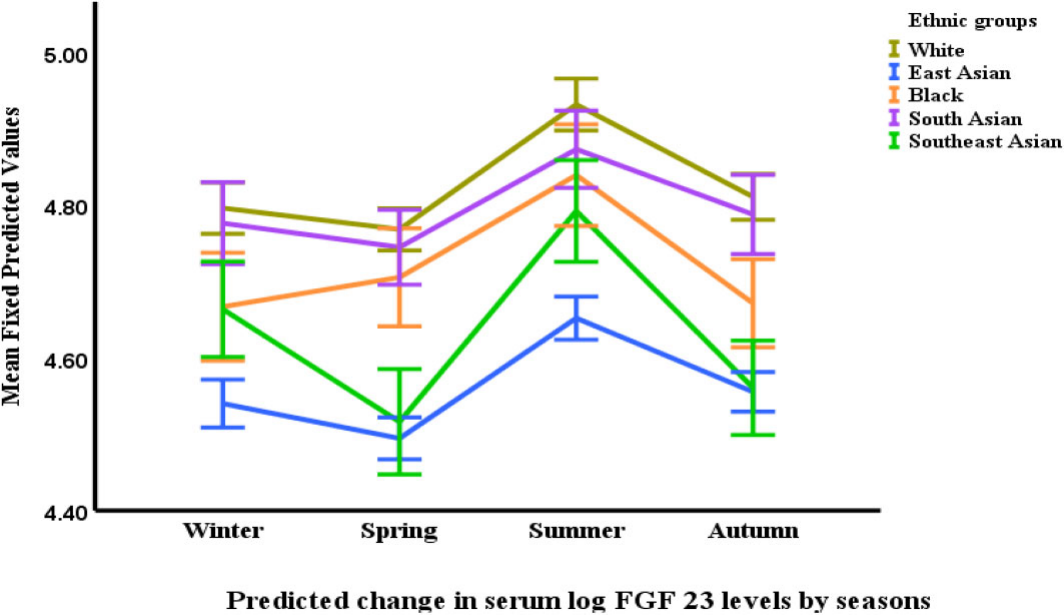
Changes in serum FGF-23^*^ by ethnicity and season. ^*^Based on mixed-effects general linear regression model predicting changes in serum natural log FGF-23 adjusted for age, gender, body surface area, visit number (as a proxy for time), eGFR, history of diabetes mellitus, use of vitamin D supplements, urine protein/creatinine ratio, serum calcium, phosphate, 25-OHD and iPTH, ethnicity, and season.

This analysis revealed significant variations in CKD-MBD marker concentrations based on participant ethnicity. White participants exhibited the highest levels of FGF-23, with concentrations 18.5% greater than Southeast Asians (*P *< .0001), 15.5% greater than East Asians (*P *< .0001), 11.1% greater than Blacks (*P *= .005) and 8.0% greater than South Asians (*P *= .034). Interestingly, South Asian participants also had elevated FGF-23 levels compared with both Southeast Asians (11.5% higher, *P *= .015) and East Asians (9.0% higher, *P *= .020) (Table 2 and Fig. 2).

**Table 2:** Serum FGF-23 and CKD-MBD marker concentrations compared between ethnic groups^a^.

	FGF-23 (pmol/L)		Calcium (mg/dL)		Phosphate (mg/dL)		25-OHD (ng/mL)		iPTH (pg/mL)	
Ethnic groups	% difference^b^ (95 % CI)	*P*	Coefficient (95% CI)	*P*	Coefficient (95 % CI)	*P*	% difference^b^ (95 % CI)	*P*	% difference^b^ (95 % CI)	*P*
Black vs White	−11.1 (−18.1 to −3.5)	**.005**	0.23 (0.16 to 0.30)	**<.0001**	−0.04 (−0.14 to 0.05)	.380	−10.0 (15.5 to− 4.0)	**.001**	43.8 (31.9 to −56.7)	**<.0001**
South Asian vs White	−8.0 (−14.7 to −0.6)	**.034**	0.07 (−0.00 to 0.13)	.053	0.10 (0.01 to 0.19)	**.027**	−20.1 (−24.7 to 15.2)	**<.0001**	11.6 (3.0 to 21.2)	**.007**
Southeast Asian vs White	−18.5 (−25.8 to −10.6)	**<.0001**	−0.03 (−0.11 to 0.05)	.506	0.05 (−0.60 to 0.15)	.392	−9.6 (−15.9 to −2.7)	**.007**	−8.3 (−17.0 to −1.2)	.085
East Asian vs White	−15.5 (−20.6 to −10.1)	**<.0001**	−0.09 (−0.15 to −0.04)	**.001**	0.13 (0.06 to 0.20)	**<.0001**	−13.8 (−17.9 to −9.5)	**<.0001**	−17.3 (−22.6 to 11.6)	**<.0001**
Black vs East Asian	5.1 (−3.6 to 14.7)	.248	0.32 (0.25 to 0.40)	**<.0001**	−0.17 (−0.27 to −0.07)	**.001**	4.4 (−2.3 to 11.6)	.204	73.8 (58.9 to −90.2)	**<.0001**
South Asian vs East Asian	9.0 (1.3 to 17.2)	**.020**	0.16 (0.09 to 0.22)	**<.0001**	−0.03 (−0.11 to 0.05)	.482	−7.3 (−12.4 to −1.9)	**.009**	35.1 (25.1 to 45.9	**<.0001**
Southeast Asian vs East Asian	−3.5 (−11.7 to 5.3)	.420	0.06 (−0.02 to 0.14)	.119	−0.08 (−0.19 to −0.02)	.111	4.9 (−2.1 to 12.4)	.173	10.7 (0.7 to 21.8)	**.034**
Southeast Asian vs Black Asian	−8.3 (−17.8 to 2.1)	.116	−0.26 (−0.36 to −0.16)	**<.0001**	0.09 (−0.04 to 0.21)	.164	0.4 (−7.7 to 9.3)	.11	−36.2 (−43.2 to −28.5)	**<.0001**
Southeast Asian vs South Asian	−11.5 (−19.8 to −2.3)	**.015**	−0.09 (−0.18 to −0.07)	**.034**	−0.05 (−0.17 to 0.06)	.365	13.2 (4.8 to 22.4)	**.002**	−18.0 (−26.2 to −8.7)	**<.0001**
South Asian vs Black	3.6 (−5.6 to 13.8)	.462	−0.17 (−0.24 to −0.08)	**<.0001**	0.14 (0.03 to 0.25)	**.010**	−11.2 (−17.5 to −4.5)	**.001**	−22.1 (−29.6 to −14.1)	**<.0001**

^a^Based on mixed-effects general linear regression also adjusted for age, gender, body surface area, visit number (as a proxy for time), eGFR, history of diabetes mellitus, use of vitamin D supplements, ethnicity, and serum calcium, phosphate, 25-OHD, iPTH or FGF-23 as appropriate.

^b^Differences in log-transformed dependent variables between ethnic groups reported as % difference calculated using: [exp(B) – 1] × 100.

Values in bold indicate statistical significance with a *P*-value < .05.

**Figure 3:**
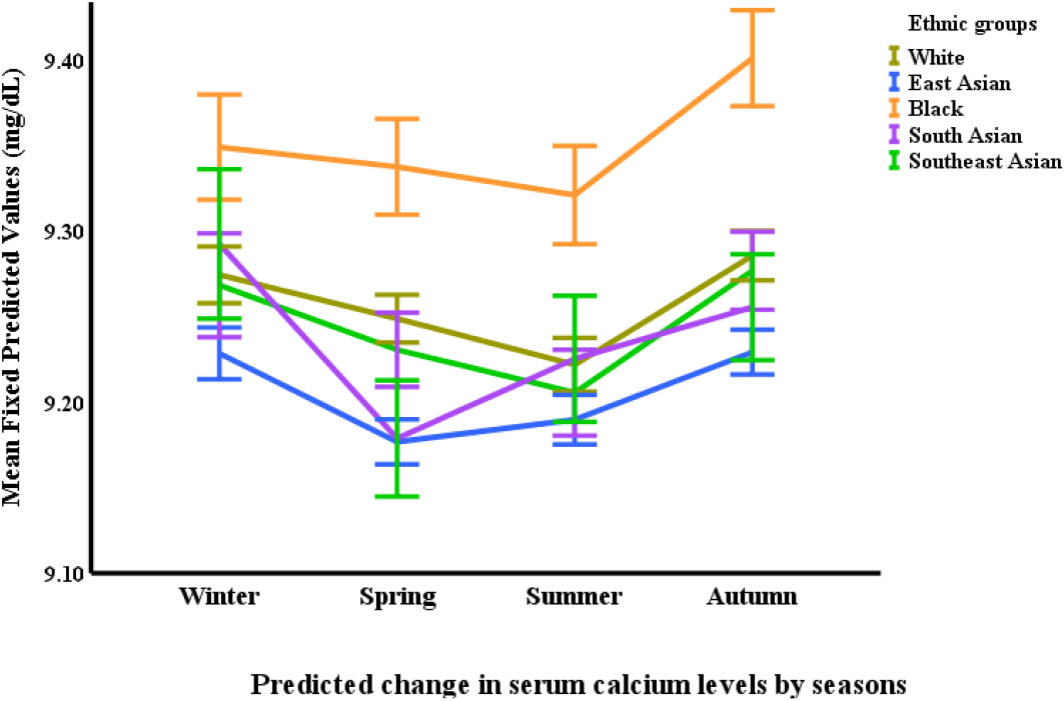
Changes in serum calcium^*^ by ethnicity and season. ^*^Based on mixed-effects general linear regression model predicting changes in serum calcium adjusted for age, gender, body surface area, visit number (as a proxy for time), eGFR, history of diabetes mellitus, use of vitamin D supplements, urine protein/creatinine ratio, serum FGF-23, phosphate, 25-OHD and iPTH, ethnicity, and season.

**Figure 4:**
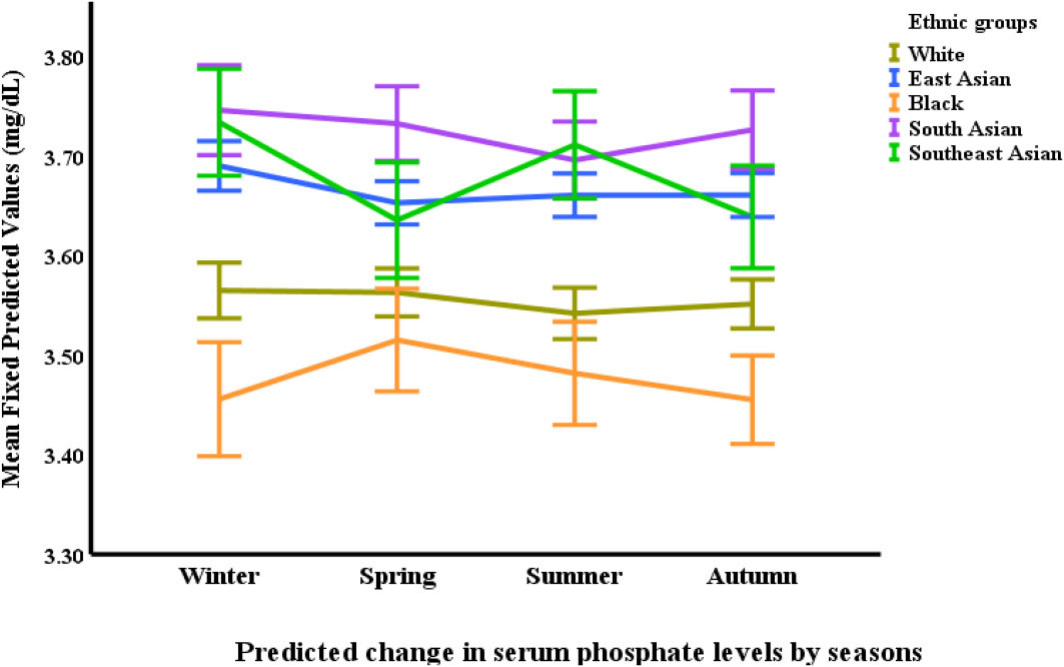
Changes in serum phosphate^*^ by ethnicity and season. ^*^Based on mixed-effects general linear regression model predicting changes in serum phosphate adjusted for age, gender, body surface area, visit number (as a proxy for time), eGFR, history of diabetes mellitus, use of vitamin D supplements, urine protein/creatinine ratio, serum calcium, FGF-23, 25-OHD and iPTH, ethnicity, and season.

Black participants demonstrated the highest serum calcium concentrations across all ethnicities. Their calcium levels were 0.32 mg/dL higher than East Asians (*P *< .0001), 0.26 mg/dL higher than Southeast Asians (*P *< .0001), 0.23 mg/dL higher than Whites (*P *< .0001) and 0.17 mg/dL higher than South Asians (*P *< .0001). Conversely, East Asian participants had lower calcium levels compared with both South Asian (0.16 mg/dL lower, *P *< .001) and White groups (0.09 mg/dL lower, *P *= .001). Phosphate levels were highest in the South Asian participant group. They displayed concentrations 0.14 mg/dL higher compared with Blacks (*P *= .010) and 0.10 mg/dL higher compared with Whites (*P *= .027). Additionally, East Asian participants had elevated phosphate levels compared with both Blacks (0.17 mg/dL higher, *P *= .001) and Whites (0.13 mg/dL higher, *P *< .0001) (Table 2 and Figs 3, 4).

Regarding vitamin D (25-OHD), White participants had the highest levels, with a statistically significant difference compared with all other ethnicities (*P *< .0001). Specifically, White participants had 20.1% higher 25-OHD levels than South Asians (*P *< .0001), 13.8% higher than East Asians (*P *< .0001), 10.0% higher than Blacks (*P *= .001) and 9.6% higher than Southeast Asians (*P *= .007). Notably, South Asian participants had the lowest 25-OHD levels despite reporting the most frequent vitamin D supplementation. Finally, Black participants exhibited the highest levels of iPTH. Their iPTH concentrations were significantly higher than all other ethnicities (*P *< .0001), with levels 73.8% greater than East Asians (*P *< .0001), 43.8% greater than Whites (*P *< .0001), 36.2% greater than Southeast Asians (*P *< .0001) and 22.1% greater than South Asians (*P *< .0001). Conversely, East Asian participants had the lowest iPTH levels compared with all other ethnicities (*P *< .0001) (Table 2).

### Seasonal variations in CKD-MBD markers

**Figure 5:**
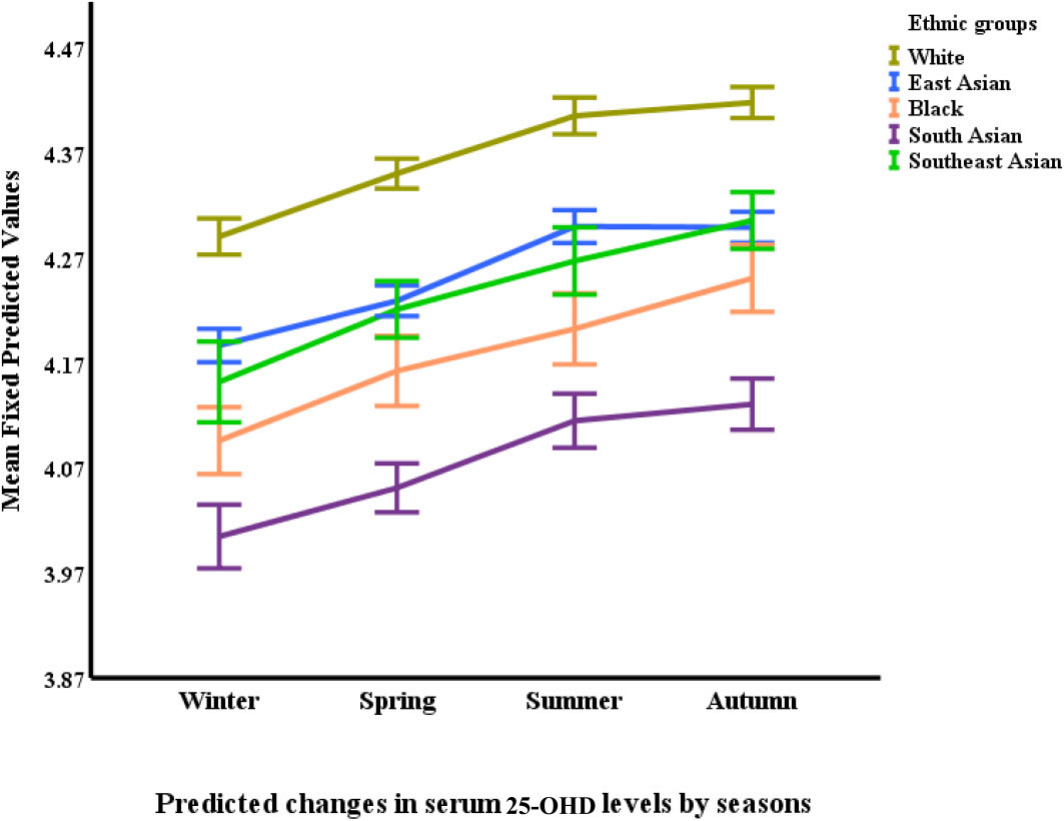
Changes in serum 25-OHD^*^ by ethnicity and season. ^*^Based on mixed-effects general linear regression model predicting changes in serum 25-OHD adjusted for age, gender, body surface area, visit number (as a proxy for time), eGFR, history of diabetes mellitus, use of vitamin D supplements, urine protein/creatinine ratio, serum calcium, phosphate, FGF-23 and iPTH, ethnicity, and season.

**Figure 6:**
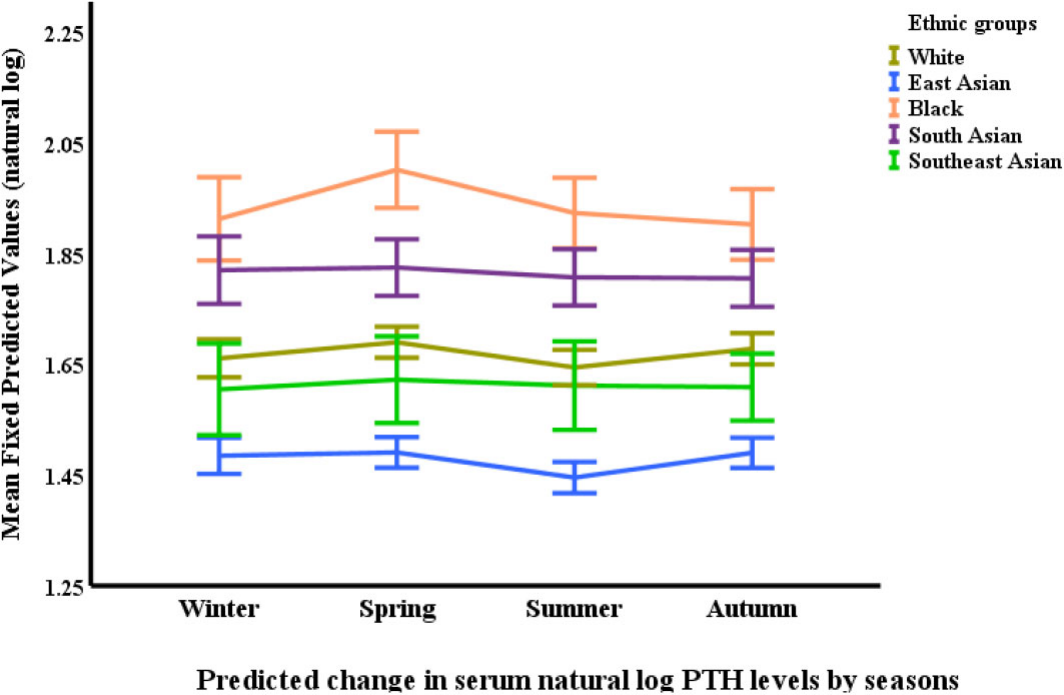
Changes in serum iPTH^*^ by ethnicity and season. ^*^Based on mixed-effects general linear regression model predicting changes in serum iPTH adjusted for age, gender, body surface area, visit number (as a proxy for time), eGFR, history of diabetes mellitus, use of vitamin D supplements, urine protein/creatinine ratio, serum calcium, phosphate, FGF-23 and 25-OHD, ethnicity, and season.

Seasonal variations were observed in some of the CKD-MBD markers. FGF-23 levels were highest in the summer compared with all other seasons. Specifically, summer FGF-23 levels were 11.9% higher than autumn (*P *< .0001), 13.2% higher than winter (*P *< .0001) and 15.5% higher than spring (*P *< .0001). Spring FGF-23 levels were also lower than autumn by 3.9% (*P *= .017). Calcium levels exhibited a distinct seasonal pattern, with the lowest concentrations observed in the summer compared with all other seasons (*P *< .0001). Summer calcium levels were statistically lower than both autumn and winter, and slightly lower than spring. Spring calcium levels were also significantly lower compared with both autumn (*P *= .008) and winter (*P *= .008) (Table 3 and Figs 2–6).

**Table 3:** Serum FGF-23 and CKD-MBD marker concentrations compared between seasons of measurement^a^.

	FGF-23 (pmol/L)		Calcium (mg/dL)		Phosphate (mg/dL)		25-OHD (ng/mL)		iPTH (pg/mL)	
Seasons	% difference^b^ (95 % CI)	*P*	Coefficient (95% CI)	*P*	Coefficient (95 % CI)	*P*	% difference^b^ (95 % CI)	*P*	% difference^b^ (95 % CI)	*P*
Spring vs summer	−15.5 (−18.6 to −12.2)	**<.0001**	2.1	**.008**	0.04 (0.01 to 0.08)	**.019**	−5.6 (−7.5 to–3.7)	**<.0001**	1.9 (−0.1 to 4.1)	.071
Autumn vs summer	−11.9 (−15.3 to −8.5)	**<.0001**	0.06 (0.04 to 0.08)	**<.0001**	0.03 (−0.01 to 0.06)	.166	0.1 (−2.1 to 1.8)	.913	3.7 (1.6 to 5.9)	**.001**
Winter vs summer	−13.2 (−16.4 to −10.0)	**<.0001**	0.06 (0.04 to 0.08)	**<.0001**	0.05 (0.01 to 0.08)	**.005**	−9.6 (−11.4 to −7.9)	**<.0001**	1.8 (− 0.1 to 3.9)	.060
Spring vs winter	−2.6 (−6.6 to 1.2)	.187	−0.03 (−0.05 to −0.01)	**.008**	−0.005 (−0.04 to 0.03)	0.760	4.5 (2.3 to 6.6)	**<.0001**	−2.6 (−6.3 to 1.2)	.187
Autumn vs winter	1.5 (−2.4 to 5.4)	.462	0.005 (−0.02 to 0.03)	.671	−0.02 (−0.06 to 0.01)	0.211	10.6 (8.4 to 12.9)	**<.0001**	1.5 (−2.4 to 5.4)	.462
Spring vs autumn	−3.9 (−7.1 to −0.7)	**.017**	−**0.03 (**−**0.05 to** −**0.02)**	**<.0001**	0.02 (−0.01 to 0.05)	0.260	−5.9 (−7.7 to −4.1)	**<.0001**	−1.7 (− 0.0 to 3.0)	.055

^a^Based on mixed-effects general linear regression also adjusted for age, gender, body surface area, visit number (as a proxy for time), eGFR, history of diabetes mellitus, use of vitamin D supplements, ethnicity, and serum calcium, phosphate, 25-OHD, iPTH or FGF-23 as appropriate.

^b^Differences between log-transformed dependent variables reported as % difference calculated using: [exp(B) – 1] × 100.

Values in bold indicate statistical significance with a *P*-value < .05.

Phosphate levels displayed a modest seasonal variation. Summer had lower phosphate levels compared with winter (*P *= .005) and spring (*P *= .019), but the difference was not statistically significant when compared with autumn. 25-OHD (vitamin D) levels were highest in the summer and autumn compared with winter and spring. Summer 25-OHD levels were significantly higher than both winter (*P *< .0001) and spring (*P *= .0001), and autumn levels were also significantly higher than both winter (*P *< .0001) and spring (*P *< .0001). Interestingly, no significant difference was observed between summer and autumn levels. Finally, iPTH (parathyroid hormone) levels exhibited a seasonal pattern with autumn showcasing the highest concentrations. Autumn iPTH levels were significantly higher than summer (*P *= .001).

## DISCUSSION

We looked for independent associations between serum concentrations of FGF-23 and CKD-MBD markers with ethnicity or season of measurement in this prospective observational cohort of 1234 multi-ethnic patients with pre-dialysis CKD living in Toronto, Canada. Relative to four other ethnic groups and three other seasons, we found that 25-OHD and FGF-23 levels were up to 20.1% and 18.5% higher in White persons, and up to 10.6% and 15.5% higher in the summer respectively. 25-OHD levels were lowest (and 25-OHD deficiency rates highest) in South Asian persons (despite taking the most vitamin D supplements) and in the winter. We also confirmed that calcium and intact PTH levels were highest in Black persons, calcium levels lowest in the summer and iPTH levels lowest in East Asian persons. These results affirm previous and add new data—particularly concerning FGF-23—that support independent variations in serum concentrations of FGF-23 and related CKD-MBD markers according to patient ethnicity and season of measurement.

Two broad sets of mechanisms could underpin these observations. First, differences in environmental factors that aggregate by ethnicity or season could influence serum concentrations. This could include differences in diet, supplement use, sun exposure behaviors and social determinants of health including income and education levels. For example, the demonstration that East Asian persons had the lowest iPTH and calcium levels—which has been reported in the general population—may be related to dietary differences [25]. A traditional Chinese diet is typically limited in milk and other dairy products, and thus compared with a dietary reference intake of 1000–1200 mg, the usual daily calcium intake is below 500 mg/day [26–28]. Similarly, the demonstration that 25-OHD levels were highest in White persons is consistent with reduced sun-protective behaviors and increased dietary vitamin D intake reported in White versus other ethnic groups, and with increased sun exposure in the summer versus other seasons [29, 30]. Future efforts could study the roles that modifiable environmental factors might play in the regulation of FGF-23 and CKD-MBD marker concentrations.

Secondly, differences in biological factors that aggregate by ethnicity or season could influence FGF-23 or CKD-MBD marker concentrations. These could include genetic and circannual factors. For example, Black persons have been shown to have a higher prevalence of variants in the gene coding for the epithelial channel TRPV 5 (A563T) (which plays an important role in regulating renal tubular calcium handling) which lead to lower urinary calcium excretion rates and higher serum calcium [31]. Similarly, more pronounced iPTH level fluctuations in response to hypocalcemia, larger parathyroid glands on autopsy and lower bone turnover in Black compared with White persons suggest that ethnic differences in iPTH production, secretion or effects may exist [32–36]. It is also possible that biological factors contributed to the observation that 25-OHD levels were lowest in persons of South Asian ethnicity, particularly since they also reported the highest intake of vitamin D supplements.

To our knowledge, the observation of a seasonal variation in FGF-23 concentrations is novel. Interestingly, sclerostin (another osteoclast-derived hormone) also displays a seasonal variance in healthy adults (although with an inverse nadir and zenith) [37]. It inhibits osteoblastic bone formation and promotes osteoclastic bone resorption, contributing to the circannual differences (with summer zeniths and winter nadirs) in bone strength [38]. Previous reports showing concordance of FGF-23 and sclerostin levels, including low FGF-23 levels in sclerostin knock-out mice, have suggested that sclerostin directly regulates FGF-23 [39]. However, these observations are inconsistent with the current study's findings of a summer zenith for FGF-23. FGF-23 is produced in multiple tissues, and it is likely—as seen in a report of sclerostin-independent extra-osseus FGF-23 production in patients with CKD—that extra-osseus production can also influence serum FGF-23 concentrations [40–43]. Case reports of cutaneous skeletal hypophosphatemia syndrome (a rare genetic disorder characterized by dysplastic skeletal lesions, congenital skin nevi and FGF-23-mediated hypophosphatemia) that showed a decrease in clinical symptoms and FGF-23 levels after removal of skin lesions provide further support for this premise [41, 44–46]. Future research might explore the amount and regulation of FGF-23 production by skin and other extra-osseus tissues; and the possible roles played by environmental factors—including sunlight exposure, temperature, diet and amount of physical activity—in the observed circannual and ethnic variations in FGF-23 levels.

### Limitations

This study has several limitations. First, 1,25-(OH)_2_D levels were not measured, and in keeping with the clinical guidelines, we resorted to measuring 25-OHD instead. Given that FGF-23 suppresses 1,25-(OH)_2_D rather than 25-OHD, we are unable to comment on this potential consequence of increased FGF-23 in White persons or during the summer months, and on whether any independent differences in 1,25-(OH)_2_D existed by ethnicity or season of measurement in our cohort. Second, the heterogeneity of calcium formulations in combination with or without vitamin D reported by some patients prevented us from including information about calcium and vitamin D supplementation due to uncertainty regarding the presence of these nutrients in these formulations. Third, we did not assess for possible environmental influences such as sun-exposure behaviors, physical activity, dietary factors, and the social determinants of health including income and education levels. Fourth, the FGF-23 assay used was unable to differentiate between full-length FGF-23 and its carboxy-terminal fragments. Given that these might have different biologic effects, future studies might determine whether the observed associations between FGF-23 and ethnicity or season of measurement are modified by type of FGF-23 assay. Fifth, although large, this was a single-center study and the results would need to be reproduced in other multi-ethnic centers to increase its generalizability. Finally, larger longer-term studies designed to detect differences in clinical outcomes relative to changes in FGF-23 and CKD-MBD markers of the observed magnitudes would be required to ascribe clinical significance to these findings.

## CONCLUSIONS

Patient ethnicity and season of measurement were independently associated with serum FGF-23 and CKD-MBD markers in this multi-ethnic cohort of patients with pre-dialysis CKD living at high latitude. Further studies of the underlying mechanisms could increase our understanding of how FGF-23 and CKD-MBD marker concentrations are regulated.

## Data Availability

The research data that support the findings of this study are not publicly available. Further inquiries can be directed to the corresponding author.
